# 

**Published:** 1884-04

**Authors:** 


### MEDICAL ASSOCIATION OF GEORGIA.

The next meeting of this Association will be held in Macon, April 16th, 17th and 18th. Below we give the programme as received from the chairman of the Committee of Arrangements, Dr. Wm. F. Holt:

### PROGRAMME.

*First Day.*—Wednesday Morning.—Convene at Court House at 11 o'clock.

OPENING EXERCISES.

Prayer by ——————. Address of welcome by Wm. F. Holt, M. D., in behalf of Committee of Arrangements. President's Annual Address. Regular Order. Adjournment at 1 o'clock.

*Afternoon.*—Convene at 3 o'clock. Regular Order. Adjournment as the Association may determine.

*Second Day.*—Thursday—Morning.—Convene at 10 o'clock. Regular Order. Orator's Address at 12 o'clock. Adjournment at 1 o'clock.

*Afternoon.*—Convene at 3 o'clock. Regular Order. Adjournment as the Association may determine.

*Evening.*—Grand Banquet at Brown's Hotel, 9 p. m.

*Third Day.*—Friday—Morning.—Convene at 10 o'clock. Regular Order. Adjournment at 1 o'clock.

*Afternoon.*—Convene at 3 o'clock. Regular Order. Adjournment as the Association may determine.

### CONTENTS.

ORIGINAL COMMUNICATIONS—

### NOTICE.

Original communications solicited. When articles require illustration the engravings will be furnished by the publishers free of charge.

Subscription: $2.50 a year.

Subscribers will please not send us money by Postal Notes. Remit by Express, Draft, Post-office Order or Registered Letter.

Address all letters and books and make all drafts payable to

The Atlanta Medical and Surgical Journal,
Care Jas. P. Harrison & Co., Atlanta, Ga.

